# Pediatrics

**Published:** 1897-01

**Authors:** Maud Josephine Frye

**Affiliations:** Clinical instructor in diseases of children, Medical Department, University of Buffalo


### Progress in Medical Science.

### PEDIATRICS.

Reported by MAUD JOSEPHINE FRYE, M. D.,
Clinical instructor in diseases of children, Medical Department, University of Buffalo.

### PEDIATRICS: PAST, PRESENT AND PROSPECTIVE.

DR. S. W. KELLEY, of Cleveland, in an address before the Ohio State Pediatric Society, reprint from *Cleveland Medical Gazette*, made an interesting attempt to solve the future of pediatrics as a specialty, at the same time giving a review of its past and its present standing. To this end he addressed letters to various teachers of pediatrics and to the deans or registrars of the sixty-five colleges in the United States and Canada. The replies he received show that at present pediatrics is taught as a separate branch in fifty schools, in connection with another subject in fifteen. While forty of the teachers interviewed regard the subject as including both the medical and surgical diseases of children, the teaching in thirty-seven colleges covers the medical side alone. In nine colleges only is the teaching entirely didactic. Replying to the inquiry as to the amount of instruction given in this branch ten years ago, the answers show that much progress has been made. Replies from thirty colleges can be analysed. In fourteen of these it was taught as now, in nine in combination with other branches, in four not at all, in three very little. Concerning its teaching twenty years ago thirty-eight schools significantly leave the question unanswered, one only reports it as taught as a separate branch, and six say it was taught but little.

Dr. Kelley further questioned his correspondents concerning the practicability, desirability and probability of the specialisation of pediatrics after the manner of gynecology. The answers received were about evenly divided, and the views of Dr. Kelley himself seem best worth quoting. He says:

I am opposed to the making of specialists in college, and always take in hand seriously the undergraduate who expresses his intention of paying particular attention to a certain branch with a view to making it a specialty when he shall have graduated. It is this kind of specialist who, as much as the advertising quack, has brought the very word into disrepute. I tell my young friend that it will be time enough when he has thoroughly grounded himself in the general principles and practice of his profession, and has had ten or fifteen years of experience, to think of devoting his time in great part to some one line more than others. I would not discourage any young practitioner from endeavoring to increase his knowledge and perfect his skill in certain particular lines or line according as he may have talent, taste or opportunity to study in that direction, for the field has become too wide for one to become expert in everything; and with quaint old Norris "I think a little plot of ground thick sown is better than a great field, which, for the most part of it, lies fallow." If bye and bye a man becomes wise or skilful beyond his fellows in a certain line of work, and they keep him so busy therein that he has no time for anything else, I can see no objection to his doing that work, whether he is called a specialist or whether he is called an expert in that line. And if all specialists were made in this way there would be no cause for complaint from anybody. In regard to pediatrics, . . . . it is probable that in all large centers of population certain doctors will become known as particularly expert in diseases of children, and whether they are called pediatrists or specialists matters little—they will be called frequently in consultations, and their practice will be largely, perhaps in some instances entirely, among children.

In regard to the progress that is likely to be made in the near future, I cannot agree with those who expect pediatrics to do no more than keep pace with the march of general medicine and surgery. The progress it has made in the past twenty years, the degree to which it has been differentiated from the other branches and separated even from those which were until recently associated with it, the number and value of recent additions to its literature, the promptness with which not only every discovery in general medicine and surgery and all specialties is tried and if practicable modified to suit the cases of children, and the zeal with which earnest workers are pursuing special investigations in this branch, all tell me that pediatrics is destined to make rapid advance in the near future. I believe it will lead out perceptibly and distinctly in the next decade, as gynecology has in the past though not to the same degree. Heaven forbid that it should be "boomed" to that extent.

---

### THE ADMINISTRATION OF ETHER TO INFANTS.

INFANTS (F. Woodhouse Braine, in the *Practitioner.—Pediatrics*) are readily brought under its influence without the slightest cyanosis. The young administrator may be puzzled at first how to estimate the degree of anesthesia produced in so young a subject. The writer has found the following method of great service: Place the index finger in the infant's hand, and it will be found that during the earlier part of the administration the finger is grasped very tightly, the palmar reflex being active; but as insensibility approaches, the infant's fingers gradually relax, and as soon as they become lissom the operation may be commenced. As in the case of adult patients our guide is the conjunctival reflex, so in the infant one should devote his constant attention to the amount of palmar and digital reflex action present. This method is preferable to the conjunctival test, because the conjunctiva of a baby loses its sensitiveness very quickly after being touched a few times with the finger. The infant may appear to be thoroughly insensible by the conjunctival test when it is actually on the point of recovering from the anesthetic, and under such conditions the reapplication of the face-piece will cause struggling, to the great inconvenience of the operator. The writer has never seen bronchitis due to ether produced in a baby, but he is exceedingly careful to guard against cold air being breathed for several hours after the operation. The opening of a window is strictly forbidden, and, when possible, the baby is not even removed from the room in which the operation has been performed for at least six hours, and he finds that when these directions are strictly adhered to there is no danger of bronchitis occurring; moreover, the tendency to sickness is greatly diminished, and this he attributes to the non-stimulation by cold air of the nerves supplying the lungs with the consequent reflex action on the stomach.

---

### USES OF THE THYROID GLAND.

Cabot (*Medical News*, September 12, 1896,) reviews the literature regarding the clinical uses of the thyroid gland. He concludes as follows: (1) Of great value in simple parenchymatous goitre, especially in young people. (2) Of considerable value to reduce weight in obesity, especially in anemic, flabby types, and provided the relapse is prevented by diet and exercise. (3) It seems to deserve a further trial in obstinate cases of psoriasis, sclerodema and lupus; also in tetany, certain phases of insanity and retarded development in children. (4) In exophthalmic goitre it rarely does good and often harm. (5) In chlorosis, rickets, diabetes and tuberculosis the evidence is not sufficient to warrant interference.

Any considerable quickening or palpitation should lead us to discontinue the drug until cardiac action is again normal. There are no dangers in the use of the drug, provided we begin with small doses, from one to two grains of American extracts, and gradually increase, watching the pulse. It should never be given to a patient who cannot be closely watched.

---

### DIPHTHERIA OF THE NASO-PHARYNX.

FREEMAN (*Medical Record*, October 31, 1896,) calls attention to the fact that the naso-pharynx may be the sole site of the membrane in diphtheria and advises routine examination of this region. The examination may be made with the rhinoscopic mirror, or through the anterior nares, or by bacteriological culture from the vault. He reports in detail four cases in which the membrane appeared in the naso-pharynx alone.

---

### THEORY TO ACCOUNT FOR MELENA.

LORANCHET (*Gazette Heb. de Med. et de Chir.*) attributes melena to the gradual chilling of the surface of the body after birth. The cold acts as a general debilitant and depressant of the nervous system ; general circulation is disturbed ; peripheral circulation is slowed ; the vaso-motor system is unbalanced. The change from the splanchnic cycle of the fetus to the cardia-pulmonary cycle of the new-born infant is interfered with, and in the struggle of the reflexes there is a reversion to the splanchnic cycle with passive congestion of the gastro-intestinal mucous membrane, which gives rise to the hemorrhage.—*Medical Record ; Archives of Pediatrics.*

---

### THYROID EXTRACT IN CHOREA.

WOLFSTEIN (*Archives of Pediatrics*, October, 1896,) reports a case of chorea treated with thyroid extract. The patient was a girl of twelve ill with a moderately severe type of the disease ; she had not improved under the routine treatment, arsenic and rest in bed. The daily excretion of urine being very small, though not otherwise abnormal, thyroid extract was given on account of its diuretic action. Improvement was immediate and wonderful. The amount of urine, previously from 200 c.c. to 465 c.c. per day, soon averaged 1,000 c.c. The choreic movements were lessened. The patient gained in weight. In a month she was discharged cured. The dose employed was five grains of the extract one, two or three times a day according to its effect on the heart.

